# GSTP1 as a senescence-associated diagnostic candidate in prostate cancer: integrative bioinformatics and functional validation

**DOI:** 10.3389/fonc.2026.1917548

**Published:** 2026-10-08

**Authors:** Houhe Li, Shuo Lu, Hua Wang, Dafu Yu, Zixiao Zhang, Shaowen Liu, Meinong Zhong, Hengjun Xiao, Ke Li

**Affiliations:** 1 Department of Urology, The Third Affiliated Hospital, Sun Yat-sen University, Guangzhou, China; 2 Department of Urology, Zhaoqing Hospital, The Third Affiliated Hospital, Sun Yat-sen University, Zhaoqing, China

**Keywords:** cell aging, glutathione s-transferase PI 1, immune microenvironment, machine learning, prostate cancer

## Abstract

**Introduction:**

Prostate cancer (PCa) is a common malignancy of the male genitourinary system with high metastatic potential and poor clinical outcomes, for which effective biomarkers and therapeutic targets remain limited.

**Methods:**

This study employed a multi-omics approach, integrating transcriptome profiles, age-related gene screening, and machine learning techniques, to analyze four GEO datasets, in order to identify key diagnostic biomarkers for prostate cancer (PCa).

**Results:**

Differentially expressed aging-related genes were identified, with enrichment analyses revealing significant involvement in PCa. Using machine learning algorithms, a robust diagnostic model centered on GSTP1 was developed, demonstrating high predictive performance. GSTP1 depletion drove multiple oncogenic phenotypes, including enhanced proliferation, migration, invasion, and the inhibition of cellular aging and DNA damage, suggesting that GSTP1 may promote cellular senescence while restraining malignant progression. Furthermore, its expression correlated with distinct immunocyte profiles, highlighting a role in shaping the PCa immune landscape.

**Discussion:**

Our findings confirm that GSTP1 functions as an aging-associated stress response gene, offering new perspectives on prostate cancer pathogenesis and showing promise for advancing therapeutic strategies that target both aging and immune associations. This study provides a comprehensive framework for leveraging aging biomarkers in precision oncology and highlights GSTP1 as a promising candidate for clinical translation in PCa management.

## 1 Introduction

Prostate cancer (PCa) persists as the most frequently diagnosed malignancy in men worldwide and represents the second leading cause of cancer-related death in population, responsible for approximately 375, 000 deaths annually (1). The disease imposes a profound dual burden: significantly diminishing patients’ quality of life and generating substantial economic strain on both families and healthcare infrastructures (2). The initial systemic management of metastatic prostate cancer, an androgen-dependent disease, hinges upon androgen deprivation therapy and androgen receptor pathway inhibitors, increasingly combined with docetaxel (3). Despite this multimodal approach, a majority of tumors inevitably progress to metastatic castration-resistant prostate cancer, representing the central therapeutic challenge in advanced disease (4). A critical and well-established risk factor is advancing age, with PCa incidence exhibiting an exponential increase in older men (5). Recent U.S. cancer statistics underscore this relationship, indicating that the lifetime probability of a prostate cancer diagnosis is 12.5%. Critically, this risk demonstrates a sharp age-dependent increase, rising to 9.0% among men aged ≥70 years, compared to only 1.8% in those aged 60–69 years (6). Autopsy studies further reveal a startlingly high prevalence of histologic PCa in elderly men, detected in 36% of Caucasians and 51% of African Americans aged 70–79 years, with frequency escalating dramatically with increasing age (7). The current status of research for biomarker discovery has been highlighted its potential for novel biomarkers that can be employed in clinical settings (8). Therefore, identifying novel biomarkers specifically associated with aging pathways holds significant promise for improving early detection accuracy and developing targeted therapeutic interventions tailored to this major risk factor.

Glutathione S-transferases are a large family of evolutionarily conserved, dimeric enzymes defined by their role in phase II detoxification. They are subdivided into eight classes (alpha, kappa, mu, omega, pi, sigma, theta, and zeta) according to their sequence homology, structural features, and substrate specificity (9). Their subcellular localization further divides them into cytosolic (alpha, mu, omega, pi, sigma, theta, and zeta), mitochondrial (kappa), and membrane-bound microsomal forms (10, 11). Among these, glutathione S-transferase pi (GSTP1) holds particular significance as the first member of the family to have its three-dimensional structure elucidated (12). While GSTP1 expression is generally low in most normal tissues, its overexpression is a common feature in numerous human carcinomas, where it frequently correlates with the development of chemotherapeutic drug resistance, advanced tumor stage, aggressive pathological features, and unfavorable patient prognosis (13). PCa presents a striking paradox regarding GSTP1. In contrast to its overexpression in many other malignancies, PCa is characterized by a frequent and early loss of GSTP1 expression. This silencing is mechanistically driven primarily by hypermethylation of the GSTP1 gene promoter region (14, 15). Immunohistochemical analyses demonstrate this loss vividly, with GSTP1 protein detectable in only 3 out of 91 prostatic adenocarcinoma samples examined (14). Indeed, GSTP1 inactivation via epigenetic silencing stands as the most prevalent somatic genetic alteration reported in PCa, occurring in over 90% of cases (16). The functional consequence of this loss, however, remains complex. Despite its silencing in the majority of PCa cases, the precise functional contribution of GSTP1 loss to prostate carcinogenesis, particularly in the context of aging, remains incompletely understood and represents a significant knowledge gap.

Addressing this gap, our study employed an integrated multi-data approach coupled with robust machine learning algorithms to systematically identify and prioritize aging-associated biomarkers central to PCa pathogenesis. This strategy comprehensively demonstrated that GSTP1 is a stress response gene related to the aging pathway and plays a crucial regulatory role in the progression of prostate cancer. Subsequent functional validation experiments using PCa cell-line models demonstrated that GSTP1 depletion promotes key oncogenic phenotypes, including increased cellular proliferation and migratory capacity, while attenuating senescence-associated phenotypes. Building on these findings, we sought to delineate how GSTP1 expression levels influence the broader tumor context, specifically its crucial relationship with the immune cell composition of the tumor microenvironment. This analysis revealed compelling associations suggesting GSTP1 status may influence immune cell infiltration and activity, thereby revealing potential implications for the development or efficacy of immunotherapeutic strategies in PCa subsets.

Collectively, our work contributes to a more nuanced understanding of PCa by elucidating the complex interplay between aging-related molecular mechanisms and tumor biology. We not only elucidate the functional significance of GSTP1 loss but also strongly nominate GSTP1 as a promising biomarker with diagnostic potential, and as a rational target for novel therapeutic approaches aimed at mitigating age-associated PCa progression.

## 2 Materials and methods

### 2.1 Data acquirement

Four publicly available microarray datasets (GSE46602, GSE60329, GSE71781, GSE79021) were downloaded from the Gene Expression Omnibus (GEO) database (https://www.ncbi.nlm.nih.gov/geo/). Probe-level expression matrices were converted to gene-level expression profiles using platform-specific annotation files. Standardized preprocessing procedures were implemented, including quantile normalization, inter-platform variation harmonization, and batch effect correction via established algorithm (**Supplementary Figures S1A, B**). Principal component analysis (PCA) plots were generated to validate the effectiveness of batch correction by comparing data distributions before and after adjustment (**Supplementary Figures S1C, D**). The aging-related gene list was obtained from the Aging Atlas database (https://ngdc.cncb.ac.cn/aging/age_related_genes), a comprehensive multi-omics resource for aging biology developed by the Chinese National Center for Bioinformation/National Genomics Data Center. All genes (**Supplementary Table S1**) in this database are collected from aging-related literature and manually annotated, with gene sets divided into ten functional sub-categories. The database includes both human and mouse genes, with each entry providing functional descriptions, supporting literature references, and KEGG pathway annotations.

### 2.2 Differential expression and functional enrichment analysis

Differential gene expression between PCa patients and healthy controls was performed using the limma R package (17). Statistical significance was determined with thresholds of | log (fold change) | ≥ 0.58 and FDR-adjusted p < 0.05. Visualization of the differentially expressed genes (DEGs) analysis was performed using the R environment, with packages ggplot2 and pheatmap used to plot the results in the form of volcano plots and heatmaps. For the heatmap, we displayed only the top 55 up-regulated and top 55 down-regulated genes ranked by absolute log_2_(fold change) to provide a clear visual representation of the most significantly altered transcripts. To elucidate the biological roles of DEGs, functional enrichment analysis was performed using the clusterProfiler R package (18). Gene Ontology (GO) analysis provided standardized annotations across three domains: cellular component, molecular function, and biological process. Kyoto Encyclopedia of Genes and Genomes (KEGG) pathway analysis integrated genomic, chemical, and systemic functional data to identify enriched biological pathways. Both analyses were conducted with default parameters, and enriched terms/pathways were filtered at p < 0.05 and false discovery rate (FDR) < 0.05.

### 2.3 Gene set enrichment analysis

GSEA (19) is a computational method that evaluates the enrichment level of predefined gene sets (e.g., from databases such as GO and KEGG) between two sample groups (e.g., disease vs. control) by calculating expression differences of genes within these sets. The analysis is performed by ranking genes based on the absolute expression differences between the two groups, followed by a statistical evaluation to determine if specific gene sets are concentrated at either extremity of this ordered list. By comparing the activity variations of gene sets between normal and tumor tissues, this method identifies biological pathways or functional modules associated with PCa.

### 2.4 Mendelian randomization analysis and machine learning framework for biomarker discovery

The IEU OpenGWAS database (https://gwas.mrcieu.ac.uk/) was used as exposure variables for MR analysis. A genome-wide association study (GWAS) of PCa (dataset ID: ieu-b-85) was selected as the outcome variable. The MR analysis utilized expression quantitative trait locus (eQTL) as instrumental variables (IVs) to investigate potential causal relationships between exposures and outcomes. To ensure rigorous IV selection and minimize bias, the following criteria were applied: Significance threshold: Variants associated with exposures were required to meet genome-wide significance (p < 5 × 10-8). Linkage disequilibrium (LD) control: A 10, 000-kb clustering window with an LD threshold of r² < 0.001 was implemented using the TwoSampleMR R package. Weak instrument exclusion: IVs with F-statistics ≤ 10 were excluded to mitigate weak instrument bias, as per the rule of thumb that F > 10 ensures sufficient statistical power. Two-sample bidirectional MR analyses were performed using the inverse-variance weighted (IVW) method as the primary approach, with sensitivity analyses including MR-Egger regression and weighted median to assess robustness. Heterogeneity and pleiotropy were evaluated using Cochran’s Q test (p < 0.05 indicates heterogeneity) and MR-Egger intercept test (p < 0.05 suggests pleiotropy). To identify key genes influencing PCa initiation, three machine learning algorithms were applied: Random Forest (RF) (20): Performed feature importance ranking based on mean decrease in accuracy. Support Vector Machine with Recursive Feature Elimination (SVM-RFE) (21): Iteratively removed the least predictive features using cross-validation. Least Absolute Shrinkage and Selection Operator (LASSO) (22) regression: Regularized regression to select non-zero coefficients corresponding to significant predictors. A Venn diagram was constructed to identify overlapping genes across all four approaches (MR, RF, SVM-RFE, LASSO). Genes intersecting all methods were designated as central core gene and prioritized for downstream functional validation.

### 2.5 Immune cell deconvolution using CIBERSORT

The relative abundances of 22 distinct immune cell types within the tissue samples were estimated using the CIBERSORT algorithm, applied to bulk RNA-seq transcriptomic profiles (23). The analysis utilized the CIBERSORTx platform’s R implementation, incorporating the LM22 signature matrix for cell type identification. Computational workflows were executed in R with dependency on ‘e1071’, ‘preprocessCore’, and ‘parallel’ libraries. Prior to deconvolution, transcriptomic profiles underwent quantile normalization via the preprocessCore package. Visualization strategies included compositional bar charts with color-coded sample annotations to display cell type distributions. Correlation matrices between immune subsets were generated using Spearman’s rho and presented as dendrogram-clustered heatmaps through the corrplot package (hclust method). Differential abundance testing between experimental groups was performed using non-parametric Wilcoxon tests, with results illustrated via ggplot2/ggpubr-generated boxplots. Statistical thresholds followed *p < 0.05, **p < 0.01, and ***p < 0.001conventions. Key parameters for reproducibility included 1000 bootstrap iterations in CIBERSORT for p-value calibration for cell type inclusion. All analytical procedures were implemented in R environment, ensuring robust quantification of tumor-immune interactions.

### 2.6 Cell line and culture

PCa cell lines (PC3, DU145) and the normal prostate cell line RWPE1 were procured from the Cell Bank of the Chinese Academy of Sciences (Shanghai, China), with authenticated short tandem repeat profiles and mycoplasma-free certification. Cells were cultured in RPMI-1640 (Gibco, USA) supplemented with 10% fetal bovine serum (FBS, Gibco, USA) under standard conditions (37 °C, 5% CO_2_). Subculturing employed 0.25% trypsin-EDTA (Gibco, USA), with a 1:3 split ratio. Cryopreservation utilized complete medium with 10% DMSO for long-term storage in liquid nitrogen.

### 2.7 Lentiviral transfection and quantitative real-time polymerase chain reaction

Lentiviral particles were produced in PCa cell lines PC3 and DU145 cells using a third-generation packaging system. Briefly, cells were seeded in 10 cm dishes at 70–80% confluency. For each dish, a transfection mixture was prepared by combining 10 μg of pLKO.1-shGSTP1 (target plasmid), 6.4 μg of pSPAX2 (packaging plasmid), and 3.6 μg of pMD2.G (envelope plasmid) with 40 μL of P3000 reagent in 1.5 mL of Opti-MEM. Separately, 41 μL of Lipofectamine 3000 was diluted in 1.5 mL of Opti-MEM. The diluted Lipofectamine was then mixed with the plasmid-P3000 complex and incubated for 20 minutes at room temperature. The mixture was added dropwise to the cells, followed by incubation for 6–8 hours before replacing the medium with fresh growth medium. Viral supernatants were harvested 72 hours post-transfection, clarified by centrifugation, and stored at -80 °C. For infection, target cells were incubated with viral supernatant supplemented with 8 μg/mL polybrene. Stable cell lines were selected using 5 μg/mL puromycin for 3 days, with daily medium renewal. For qRT-PCR validation, total RNA extracted using TRIzol (Thermo Fisher Scientific, USA) was reverse-transcribed with RevertAid (Vazyme, China). SYBR Green-based qPCR (Accurate Biology, China) on Roche LightCycler 480 used GAPDH-normalized primers: GSTP1 (F: AATTCTGTGACCCCCACTGAC, R: TTTGGCTGCTTTCTCCTTGC) and GAPDH (F: GAGAAGGCTGGGGCTCATTT, R: GTCAAAGGTGGAGGAGTGGG). Protein validation via Western blot followed standard protocols.

### 2.8 Western blot analysis

Total proteins were extracted from cellular using RIPA lysis buffer (Thermo Fisher Scientific, USA) containing 1% protease inhibitor cocktail to ensure protein integrity. Following centrifugation of the lysates at 12, 000 rpm for 10 minutes at 4 °C, the supernatants were collected. The protein concentration in these supernatants was then quantified using a BCA assay kit (Beyotime, China). Equal amounts of protein (30 μg/lane) were separated by 10% sodium dodecyl sulfate-polyacrylamide gel electrophoresis at constant voltage (120 V) until the dye front reached the bottom of the gel. Proteins were subsequently transferred onto polyvinylidene fluoride (PVDF) membranes (Millipore, USA) using a wet electroblotting system (Bio-Rad, USA) at 80 V for 40 minutes. Membranes were blocked with 5% non-fat dry milk in PBST (phosphate-buffered saline, 0.1% Tween-20) buffer for 1 hour at room temperature with gentle shaking. After blocking, membranes were incubated overnight at 4 °C with primary antibodies: rabbit polyclonal anti-GSTP1 (1:1000 dilution, 15902-1-AP, Proteintech, China) and rabbit monoclonal anti-GAPDH (1:2000 dilution, 14094-1-AP, Proteintech, China). Following three 10-minute washes in PBST, membranes were incubated with HRP-conjugated goat anti-rabbit secondary antibodies (1:5000 dilution, AS014, Abclonal, China) for 1 hour at room temperature. After additional washing steps, protein bands were developed using enhanced chemiluminescence (ECL) reagent (Vazyme, China) and visualized with a Tanon Imaging System. After the exposure is completed, the membrane is gently incubated in a shaker with a stripping buffer (CW0056M, CWBio) for 15 minutes, and then sealed again. After washing with the detergent, add the secondary antibody again: rabbit polyclonal anti-P21 (1:1000 dilution, 10355-1-AP, Proteintech, China), rabbit Polyclonal anti-P16 (1:1000 dilution, 10883-1-AP, Proteintech, China). After incubation is completed, the operation steps are the same as before.

### 2.9 Cell-counting kit 8 assay

PCa cell lines were allocated into negative control (sh-NC) group and GSTP1-targeting shRNA group. Cells were seeded at an optimized density of 1×10³ cells/well (96-well plate format) in 100 μL complete medium and incubated overnight at 37 °C under 5% CO_2_ to ensure adherence. At experimental initiation (0 h), culture medium was gently aspirated, and cells were washed twice with 200 μL phosphate-buffered saline (PBS, pH 7.4) to remove non-adherent debris. Fresh RPMI-1640 medium (100 μL/well) supplemented with 10% FBS was then added to maintain physiological conditions. CCK-8 reagent (10 μL/well; Dojindo, Japan) was introduced at 0, 24, 48, 72 and 96 h post-treatment. Following 2 h incubation at 37 °C, absorbance was measured at 450 nm using a BioTek Synergy H1MF (BioTek Instruments, USA). All experiments were performed in technical triplicates, with data normalized to 0 h baseline values for proliferation analysis.

### 2.10 Transwell migration and invasion assay

Cell migration and invasion capacities were evaluated using 24-well Transwell inserts (8.0 μm pore size; Corning, USA). For invasion assays, inserts were pre-coated with 50 μL Matrigel (1:8 dilution in serum-free RPMI-1640; BD Biosciences, USA) and incubated at 37 °C for 2 h to form a reconstituted basement membrane. Uncoated inserts served as migration controls. Prior to assays, cells were serum-starved for 12 h in RPMI-1640. Suspensions were prepared at 1×105 cells/mL in serum-free medium, and 200 μL aliquots were added to upper chambers. Lower chambers contained 600 μL RPMI-1640 with 10% FBS as a chemoattractant. Following 24 h incubation at 37 °C, non-migratory/non-invasive cells on the upper membrane surface were removed using cotton swabs. Migrated/invaded cells on the lower surface were fixed with 4% paraformaldehyde (Servicebio, China) for 20 min, stained with 0.1% crystal violet (Beyotime, China) for 10 min, and rinsed gently with PBS. Stained cells were imaged at 100× magnification using a Nikon microscope (Ti2-U, Japan).

### 2.11 Scratch wound healing assay

Scratch wound healing assays were conducted to evaluate migratory capacity in shRNA-transfected PCa cells (sh-NC and sh-GSTP1 groups). Cells were seeded at 1×10^5^ cells/well in 6-well plates and cultured for 24 h at 37 °C under 5% CO_2_ to achieve ~90% confluence. Using a sterile 10 μL pipette tip, uniform linear wounds were created perpendicular to the cell monolayer. Debris was removed by two gentle washes with PBS, followed by incubation in RPMI-1640 medium containing 1% FBS to minimize proliferative effects. Wound closure progression was monitored through Nikon microscope (Ti2-U, Japan) at 0 h and 24 h post-wounding.

### 2.12 Colony formation assay

Cells were seeded in 6-well plates at optimized densities (1000 cells/well) and cultured for 7 days under standard conditions to permit colony development. Following incubation, cellular monolayers were gently washed with PBS and fixed with 4% paraformaldehyde (Servicebio, China) for 20 min at room temperature. Fixed colonies were stained with 0.1% crystal violet solution (Beyotime, China) for 10 min with gentle rocking to ensure uniform dye penetration. Stained colonies were digitized using a Nikon microscope Imaging System (Ti2-U, Japan), with images captured in TIFF format for subsequent analysis.

### 2.13 Detection of doxorubicin-induced stress-induced premature senescence by senescence-associated β-galactosidase staining

For senescence induction, PC3 and DU145 cells were plated in triplicate (5 × 104 cells/well, 6-well plate) and exposed to 50 nM Doxorubicin (DOX) for a period of three days. Subsequently, the medium containing DOX was removed, then the cells were washed with 1X PBS, followed by incubation for two days in the drug-free fresh medium at 37 °C under 5% CO2 culture conditions. SA-β-gal staining kit (Beyotime, Shanghai, China) was used to detect the senescence of PCa. We performed the staining according to the manufacturer’s protocol. Briefly, after washing cells three times with PBS, fixation was carried out at room temperature for 15 min using 1 mL of the provided fixative. Following fixation, the solution was aspirated and cells were washed thrice with PBS (3 min each). Cells were then incubated with 1 mL of the working staining solution (prepared by mixing 10 μL of solution A, 10 μL of solution B, 930 μL of solution C, and 50 μL of X-Gal solution). The plate was sealed and incubated at 37 °C in a non-CO2 incubator for 12 h. After removing the staining solution and a final PBS wash, cells were visualized and quantified under an optical microscope.

### 2.14 Immunofluorescence

Cells were seeded in 6-well plates at an appropriate density and incubated overnight at 37 °C in a humidified 5% CO2 incubator to allow for cell adhesion. After 24 hours, cells were treated with doxorubicin (DOX) at a specified concentration (50 nM) for the indicated duration (48h). Following treatment, cells were gently washed twice with PBS to remove residual medium and unbound drug. Cells were then fixed with 4% paraformaldehyde in PBS at room temperature (RT) for 20 minutes. Fixed cells were washed three times with PBS (5 minutes each) to remove residual paraformaldehyde. For nuclear protein staining, cells were permeabilized with 0.1–0.5% Triton X-100 in PBS for 10 minutes to allow antibody access to the nucleus. Permeabilized cells were washed three times with PBS. Non-specific antibody binding was minimized by blocking with 5% bovine serum albumin (BSA) in PBST at RT for 1 hour. Cells were incubated with a primary antibody targeting γH2AX (1:800 dilution; Abclonal, China) diluted in 1% BSA/PBST overnight at 4 °C. After primary antibody incubation, cells were washed three times with PBST (5 minutes each). Cells were then incubated with a fluorescently labeled secondary antibody (594-Goat Anti-Rabbit Recombinant Secondary Antibody, 1:1000 dilution; Proteintech, China) in 1% BSA/PBST at RT for 1 hour in the dark. All subsequent steps were performed under low light to prevent fluorescence quenching. Nuclei were stained with 4’, 6-diamidino-2-phenylindole (DAPI, 1:1000 dilution in PBS) at RT for 5 minutes. Cells were washed three times with PBS to remove unbound DAPI. Fluorescence images were acquired using a widefield fluorescence microscope (Ti2-U, Japan). For quantitative comparison, all imaging parameters (laser intensity, exposure time, gain, and pixel resolution) were kept consistent across shNC and sh-GSTP1 samples.

### 2.15 Statistical analysis

Data were processed and analyzed with R and GraphPad Prism 8. Group comparisons employed the Wilcoxon rank-sum test or t-test for continuous variables, and the Kruskal-Wallis test for non-parametric data. Pearson’s coefficient was applied to assess bivariate relationships. Experimental results (mean ± SD; ≥3 biological replicates) were considered significant at p < 0.05 (*p < 0.05, **p < 0.01, ***p < 0.001, ns: not significant).

## 3 Results

### 3.1 Differential expression reveal key aging-associated genes in PCa

To pinpoint key molecular features in PCa, we employed the limma R package for differential expression analysis. A comparison between tumor and normal tissue profiles revealed 603 robust DEGs meeting our statistical significance criterion. We selected 55 upregulated genes and 55 downregulated genes, and generated a heatmap representation (**Figure 1A**). Visualization in a volcano plot (**Figure 1B**) reveals these DEGs, which were identified through a comparative analysis of tumor versus normal tissue expression profiles. To further explore the underlying biological mechanisms, GSEA was conducted on the dataset. GSEA identified the top five significantly enriched pathways, including Focal adhesion (**Figure 1C**) and one of them was enriched in associated with PCa (p<0.001; **Figure 1D**). To further identify the hub DEGs distinguishing the PCa group from the control group, we employed a Venn diagram to intersect the full list of 603 DEGs with aging-related genomic datasets from the Aging Atlas database. This integrative approach led to the identification of 26 overlapping hub genes (**Figure 1E**), which served as the starting candidates for subsequent machine-learning-based feature selection.

**Figure 1:**
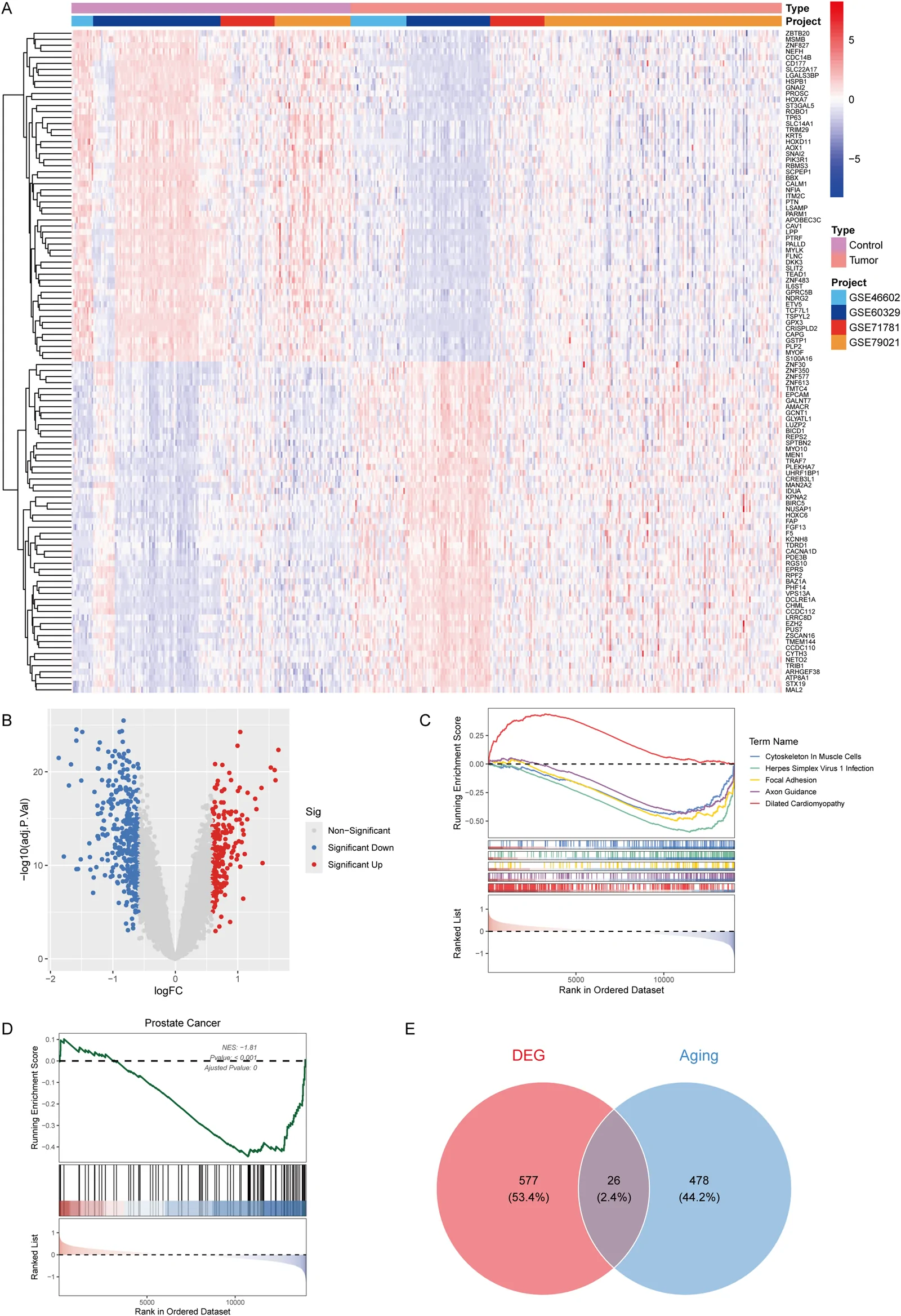
Profiling of differentially expressed genes in PCa. **(A)** Comparative visualization of DEGs expression across tumor and normal groups in a heatmap. **(B)** Graphical representation of DEGs in a volcano plot (red: up-regulated; blue: down-regulated). **(C)** GSEA results indicating the top 5 enriched gene sets for the identified DEGs. **(D)** GSEA enrichment analysis in PCa. **(E)** Venn diagram showing the overlap between differentially expressed genes and aging-related genes.

### 3.2 Functional enrichment analysis reveal key regulatory networks in PCa

Functional enrichment analysis of hub DEGs in PCa revealed multilevel regulatory networks through integrated GO and KEGG pathway analyses. GO annotation demonstrated significant enrichment across three functional domains: biological processes showed predominant involvement in epithelial cell proliferation regulation, apoptotic signaling modulation, osteoblast and lymphocyte differentiation, and neuron apoptosis; cellular component analysis highlighted localization to platelet alpha granules, secretory granule lumens, and cytoplasmic vesicle compartments; molecular function assessment identified key roles in kinase regulatory activity, insulin/cytokine receptor binding, and integrin-mediated cell adhesion (**Figures 2A, B**). KEGG pathway analysis revealed 10 significantly enriched cancer-related networks, with the PCa pathway (hsa05215) emerging as the most prominent (**Figures 2C, D**). Pathview visualization demonstrated systemic activation of this pathway, characterized by significant downregulation of tumor suppressor genes GSTP1 (impaired carcinogens) and p21 (disrupted cell cycle progression) (**Figure 2E**). This mechanism dysregulation - compromising cellular defense against carcinogens while disrupting cell cycle checkpoints - suggests an aging-related molecular mechanism driving PCa progression. These findings provide a comprehensive framework linking molecular alterations to pathway activation and cancer hallmarks in prostate tumorigenesis.

**Figure 2:**
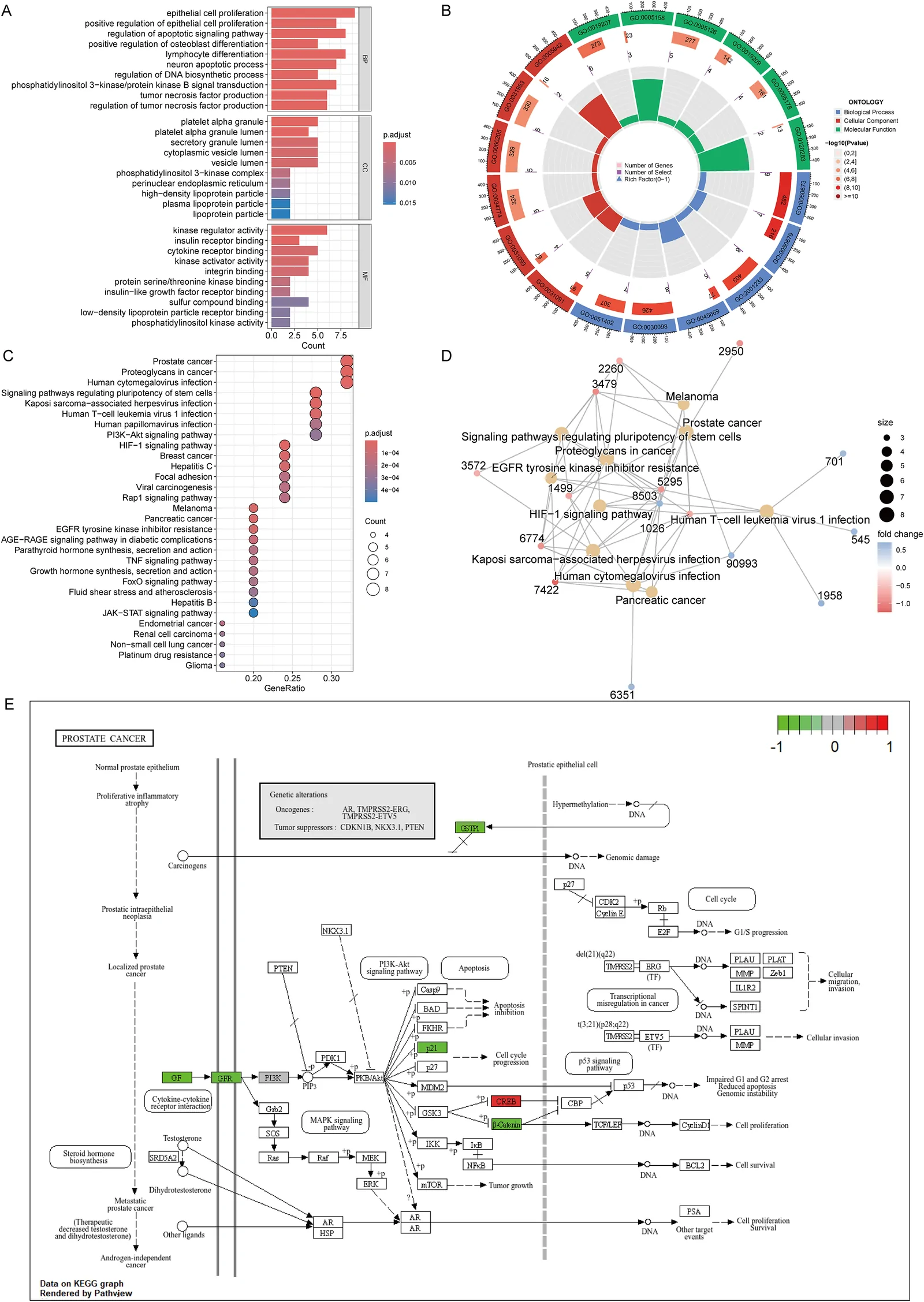
Key regulatory networks in PCa identified by functional enrichment analysis. **(A)** Bar plot illustrating significantly enriched Gene Ontology (GO) terms based on DEGs (p < 0.05). **(B)** Circular plot representing the enriched GO terms (p < 0.05). **(C)** Bubble plot of enriched Kyoto Encyclopedia of Genes and Genomes (KEGG) pathways (p < 0.05). **(D)** Network diagram of the enriched KEGG pathways (p < 0.05). **(E)** Pathview visualization demonstrated systemic activation of PCa pathway.

### 3.3 Machine learning-driven identification of diagnostic hub genes

We integrated three complementary feature selection approaches—LASSO regression, SVM-RFE, and RF—to pinpoint a more focused gene set from the initial 26 candidate core genes. LASSO regression identified 16 candidate features with 10-fold cross-validation (**Figures 3A, B**). SVM-RFE achieved superior discriminative capacity (accuracy = 0.834) through radial kernel optimization, prioritizing 11 signature genes (**Figures 3C, D**). RF analysis ranked candidates by Gini importance, retaining the top 20 features (**Figures 3E, F**).

**Figure 3:**
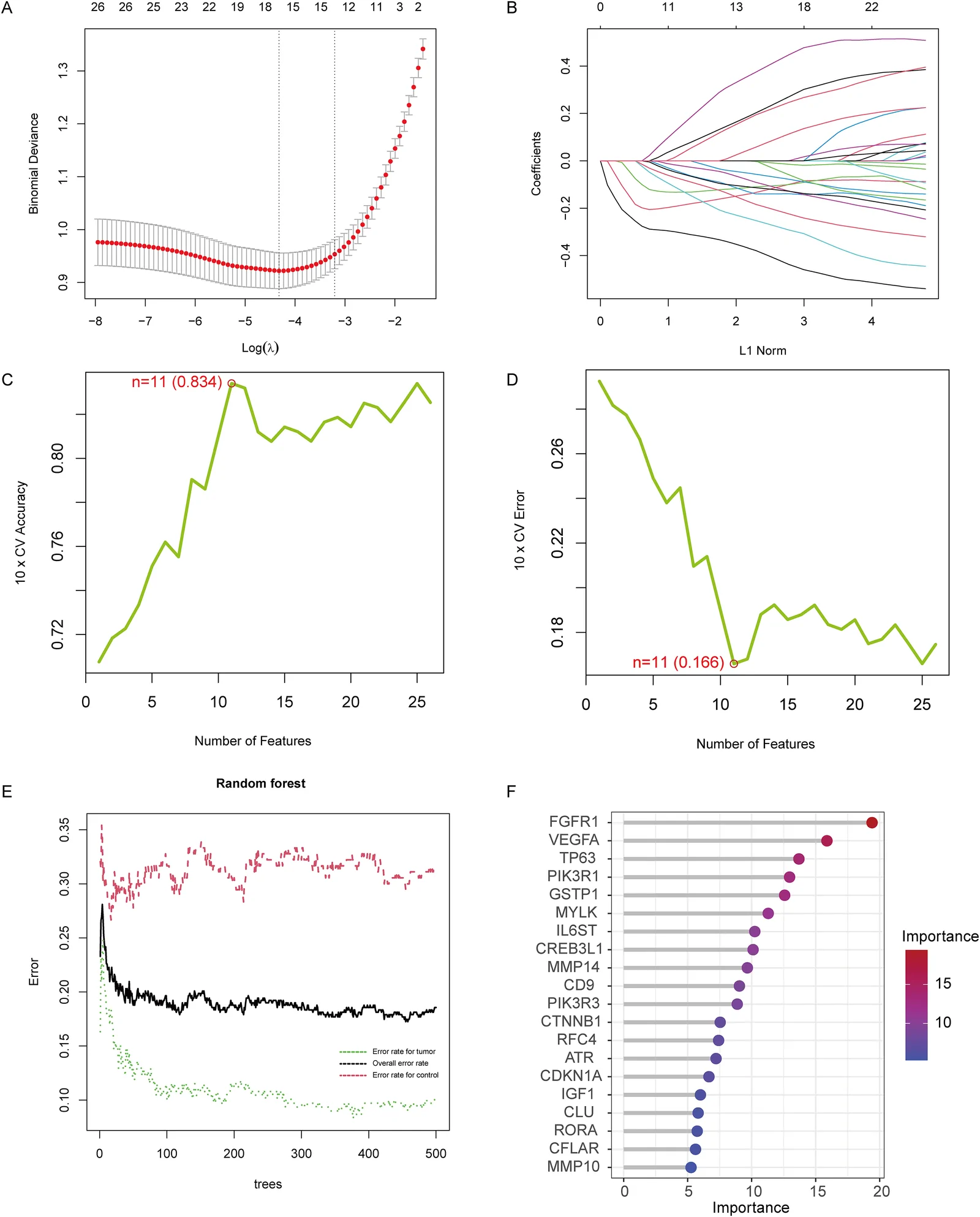
Development and performance assessment of three machine learning models. **(A)** LASSO model: cross-validation results for identifying the optimal lambda. **(B)** LASSO model: trajectory of regression coefficients across lambda values. **(C)** SVM model: cross-validation curve for model accuracy. **(D)** SVM model: cross-validation curve for classification error. **(E)** RF model: visualization of the model architecture. **(F)** RF model: bubble plot displaying ranked feature importance.

### 3.4 Mendelian randomization analysis and core biomarker identification

The forest plot (**Figure 4A**) demonstrated a negative association between GSTP1-related SNPs and PCa risk (combined effect size < 0), indicating GSTP1 as a protective factor. The funnel plot (**Figure 4B**) exhibited symmetrical distribution of SNPs, suggesting minimal small-study bias. Leave-one-out sensitivity analysis confirmed robustness of the causal estimate, with no single SNP disproportionately influencing the overall effect (**Figure 4C**). The scatter plot (**Figure 4D**) visualized the inverse relationship between GSTP1 expression and PCa risk across five regression algorithms. A downward trend in SNP effect sizes (x-axis: GSTP1-associated SNPs; y-axis: PCa risk) confirmed the dose-dependent protective effect of GSTP1. Statistical validation showed no horizontal pleiotropy (Egger intercept p = 0.988) and no significant outliers (MR-PRESSO global test p = 0.358 for GSTP1), reinforcing the reliability of our MR findings (**Supplementary Table S2**). Using IVW analysis as the primary method, we identified GSTP1 as a statistically significant protective factor (OR = 0.901, 95% CI: 0.818–0.992, p < 0.05) (**Figure 4F**). Venn diagram intersection analysis (**Figure 4E**) revealed GSTP1 as the sole gene consistently identified across all computational approaches (MR, RF, SVM-RFE, LASSO; **Supplementary Table S3**). This multi-tiered convergence underscores the biological relevance of this regulator in PCa aging-related pathophysiology.

**Figure 4:**
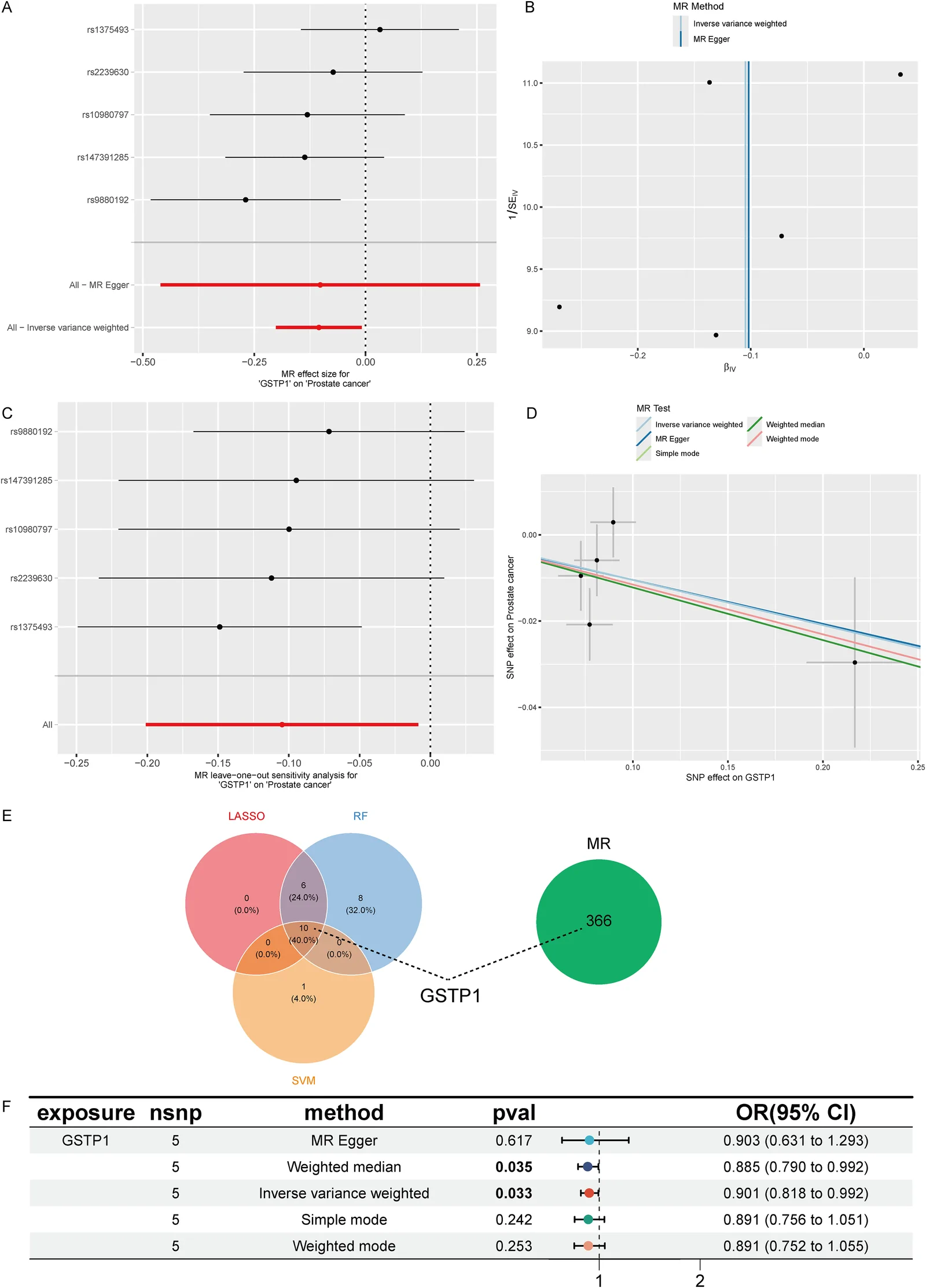
Identification of core predictive biomarkers for PCa. **(A)** Forest plot illustrating the inerse association between GSTP1-related SNPs and PCa risk. **(B)** Funnel plot displaying symmetrical distribution of SNPs. **(C)** Leave-one-out sensitivity analysis validating robustness of the causal estimate. **(D)** Scatter plot depicting the inverse correlation between GSTP1 expression levels (x-axis) and PCa risk (y-axis). **(E)** Venn diagram showing overlapping genes identified as predictive biomarkers by LASSO, SVM, and RF models. **(F)** IVW method identifying GSTP1 as a significant protective factor against PCa (p < 0.05), with the OR and 95% confidence interval shown.

### 3.5 Validation of hub prognostic biomarkers

To validate reproducibility, cross-platform analysis of GEO datasets confirmed the dysregulated expression signature: GSTP1 was significantly downregulated (p < 0.001), consistent with our primary findings (**Figure 5A**). ROC analysis of individual biomarkers revealed strong classification potential, with AUC values of 0.779 for GSTP1 (**Figure 5B**). By integrating individual gene scores, we derived a composite PCa risk score. Decision curve analysis validated the clinical utility of this score across a range of threshold probabilities (**Figure 5C**), while calibration plots showed strong concordance between model predictions and actual outcomes (**Figure 5D**). Furthermore, based on computational feature rankings, we developed a prognostic nomogram that incorporates these key biomarkers (**Figure 5E**). Taken together, our results position GSTP1 within a mechanistically grounded biomarker panel for PCa risk stratification, justifying its selection for in-depth computational and experimental validation.

**Figure 5:**
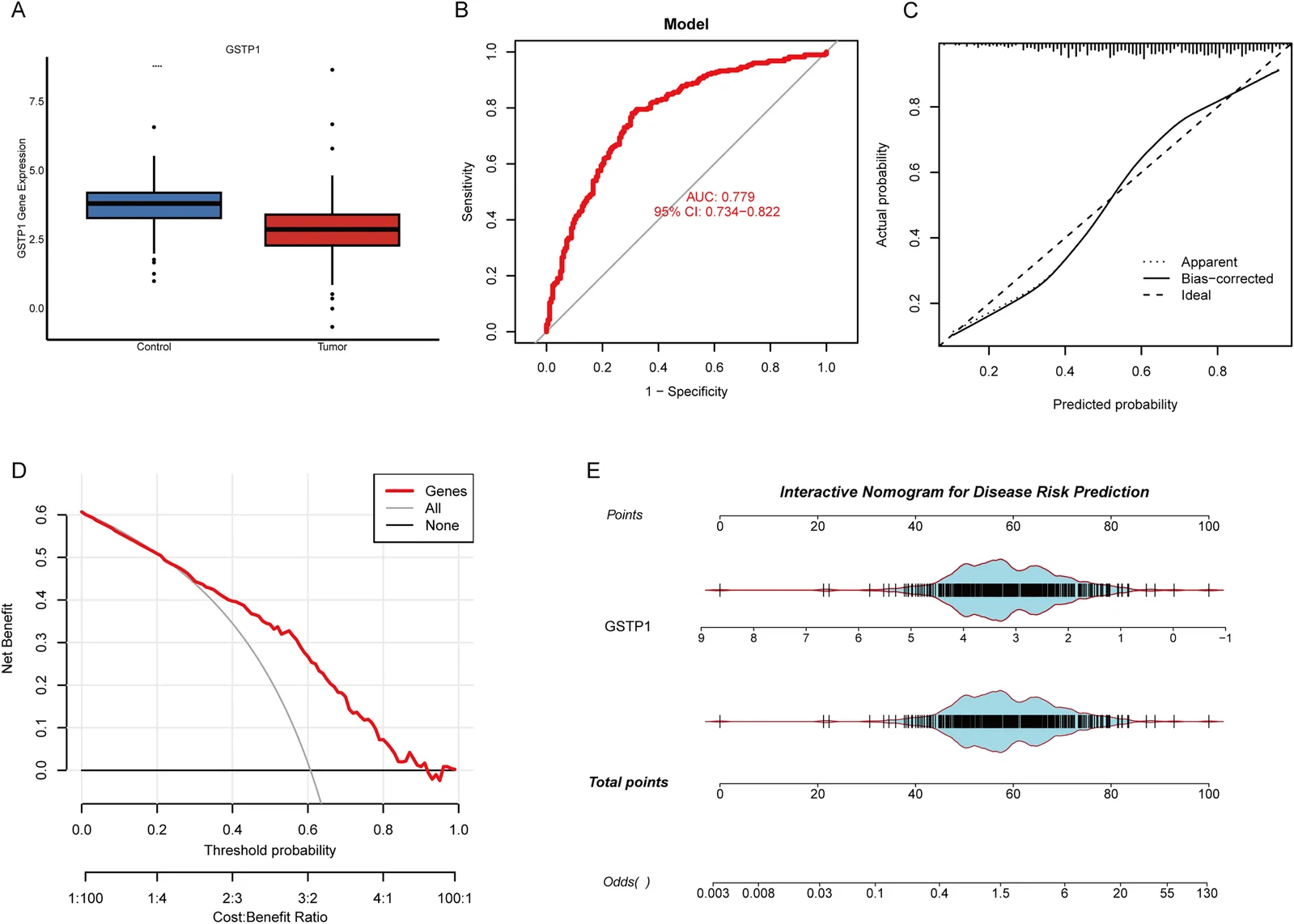
Assessment of core predictive biomarker. **(A)** Independent transcriptional validation of candidate genes (GEO database). **(B)** ROC analysis summarizing the diagnostic performance of core biomarker. **(C)** Calibration plot evaluating the model’s predictive accuracy. **(D)** Decision Curve Analysis (DCA) quantifying the clinical utility across decision thresholds. **(E)** Clinical nomogram incorporating the core biomarker for outcome prediction.

### 3.6 Analysis of the immune microenvironment composition in PCa

Emerging evidence underscores the critical involvement of immune microenvironment dynamics in PCa pathogenesis. To corroborate these findings, we employed the CIBERSORT algorithm to deconvolute the bulk transcriptomic data, thereby estimating the relative proportions of 22 distinct immune cell populations within the samples (**Figure 6A**). This systematic profiling revealed significant variations in immune infiltration patterns associated with GSTP1 expression levels. Spearman correlation analysis further revealed significant interconnections among most immune cell populations, indicating complex regulatory dynamics within the tumor microenvironment (**Figure 6B**). Our analysis revealed a general suppression of adaptive immunity within PCa tissues. Tumor samples displayed a marked depletion of B cells naive, plasma cells and T cells CD8 compared to controls. Conversely, B cell memory, T cell regulatory, T cells gamma delta, and macrophages M2 were conversely enriched (p < 0.05; **Figure 7A**). It is worth noting that GSTP1 has a significant correlation with tumor immunity: it correlated positively with anti-tumor immune components (e.g., B cells naïve, Plasma cells, T cells CD8, NK cells resting and monocytes) and negatively with multiple pro-tumorigenic populations (e.g., B cell memory, T cells CD4 memory activated, T cells regulatory, T cell gamma delta, macrophages M2, Dendritic cells activated and eosinophils **Figures 7B, C**; **Supplementary Figure S2**). This pattern suggests a dual role for GSTP1 in promoting effector T cell function while potentially restraining immunosuppressive checkpoint activity. The identified immunophenotypic associations position GSTP1 as a potential associate factor of tumor-immune crosstalk, revealing a possibly novel mechanistic insights into its tumor-modulatory functions. These findings substantiate the therapeutic relevance of GSTP1 as a potential immunotherapeutic target in PCa management, warranting further investigation into its precise regulatory mechanisms and clinical translatability.

**Figure 6:**
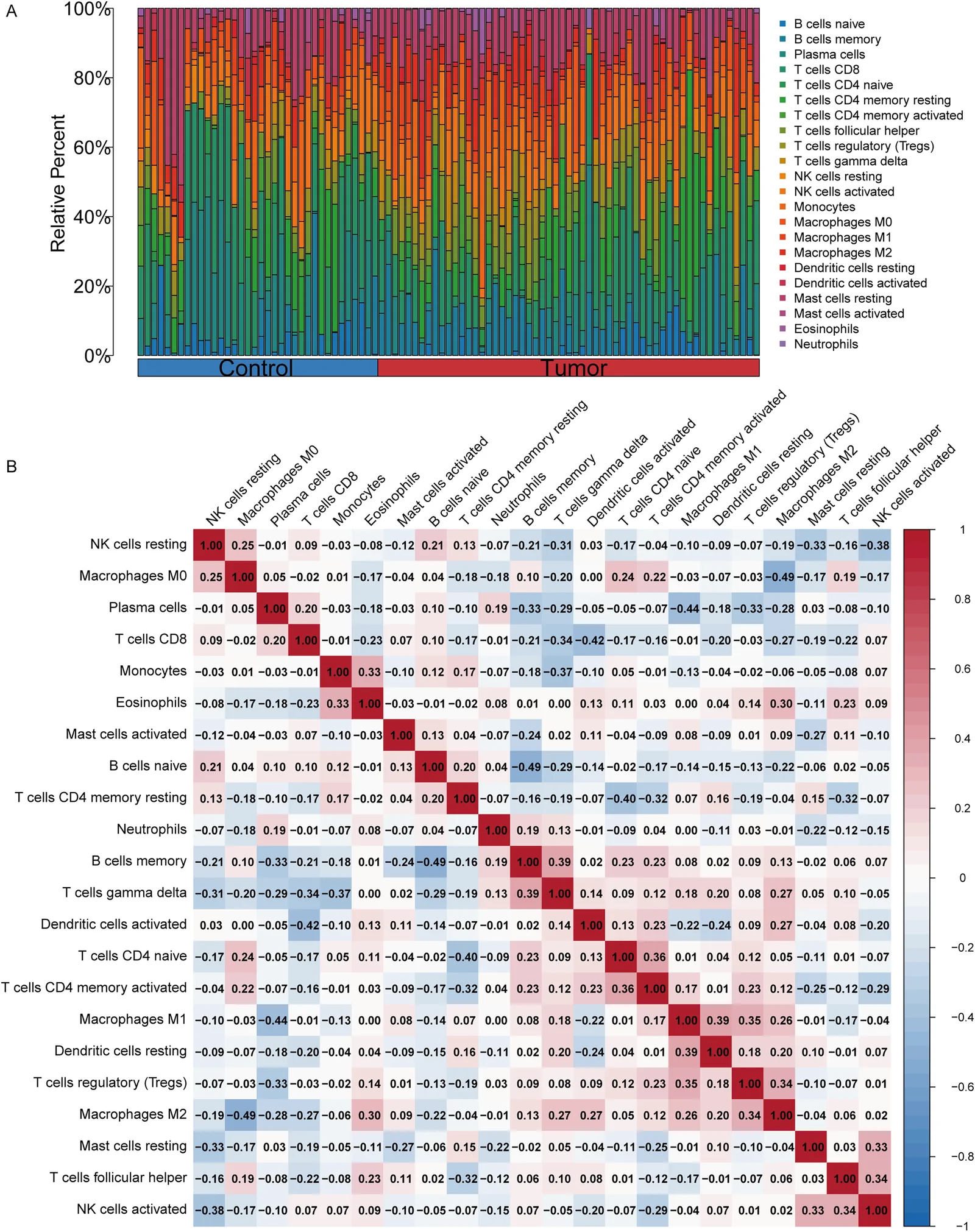
Immune microenvironment characterization via CIBERSORT. **(A)** Relative abundance of 22 immune cell types across samples. **(B)** Heatmap depicting correlative relationships among the infiltrating immune cell populations.

**Figure 7:**
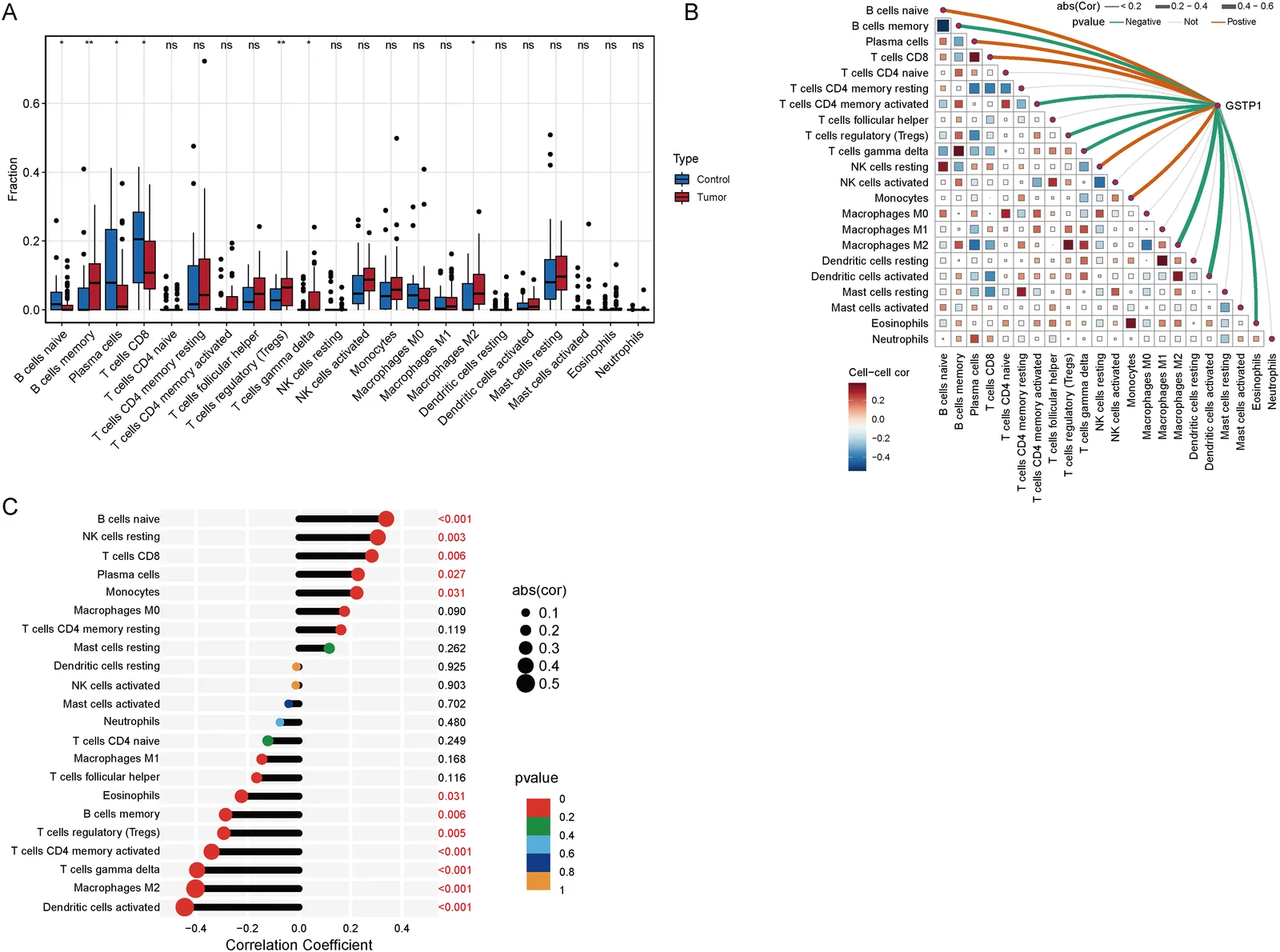
Characterization of tumor immune microenvironment and drug enrichment analysis. **(A)** Box plots comparing immune cell infiltration scores between Tumor and Control groups, with statistical significance (p < 0.05). **(B)** LinkET plot visualizing the correlation network between GSTP1 expression levels and immune cell subsets, where node size represents correlation strength and color indicates directionality. **(C)** Lollipop plot showing Spearman’s rank correlation coefficients between GSTP1 and immune cell types.

### 3.7 GSTP1 depletion promotes PCa cell line proliferation, metastasis and aging progression

We therefore sought to experimentally define the contribution of GSTP1 to PCa malignancy through a set of *in vitro* functional studies. we first evaluated its endogenous expression levels in two widely used PCa cell lines, PC3 and DU145. WB analysis and qRT-PCR consistently demonstrated significantly downregulated GSTP1 expression in the two PCa cell lines when benchmarked against the normal prostate epithelial controls (RWPE-1 cells; **Figure 8A**). To silence GSTP1 via RNAi, we deployed two specific shRNAs (sh-GSTP1–1 and sh-GSTP1-2), using a non-targeting shRNA (shNC) as control. WB analysis validated potent GSTP1 knockdown by both targeting shRNAs (**Supplementary Figure S3A**). This loss of GSTP1 drove a pro-tumorigenic phenotype, significantly boosting both cell proliferation and colony formation capacity compared to the non-targeting shNC (**Figures 8B, C**). In scratch wound healing assay, GSTP1-deficient cells exhibited significantly enhanced migratory capacity at 12 hours compared to controls (**Figure 8D**). Transwell assays further revealed concurrent enhancement of both migration and invasion, with sh-GSTP1–1 and sh-GSTP1–2 groups showing more migrated or invaded cells compared to controls (**Figure 8E**). Taken together, our data establish that GSTP1 acts as a tumor suppressor on key oncogenic processes—proliferation, migration, and invasion—in PCa cells. Its loss disrupts aging-associated homeostasis, thereby promoting malignant progression.

**Figure 8:**
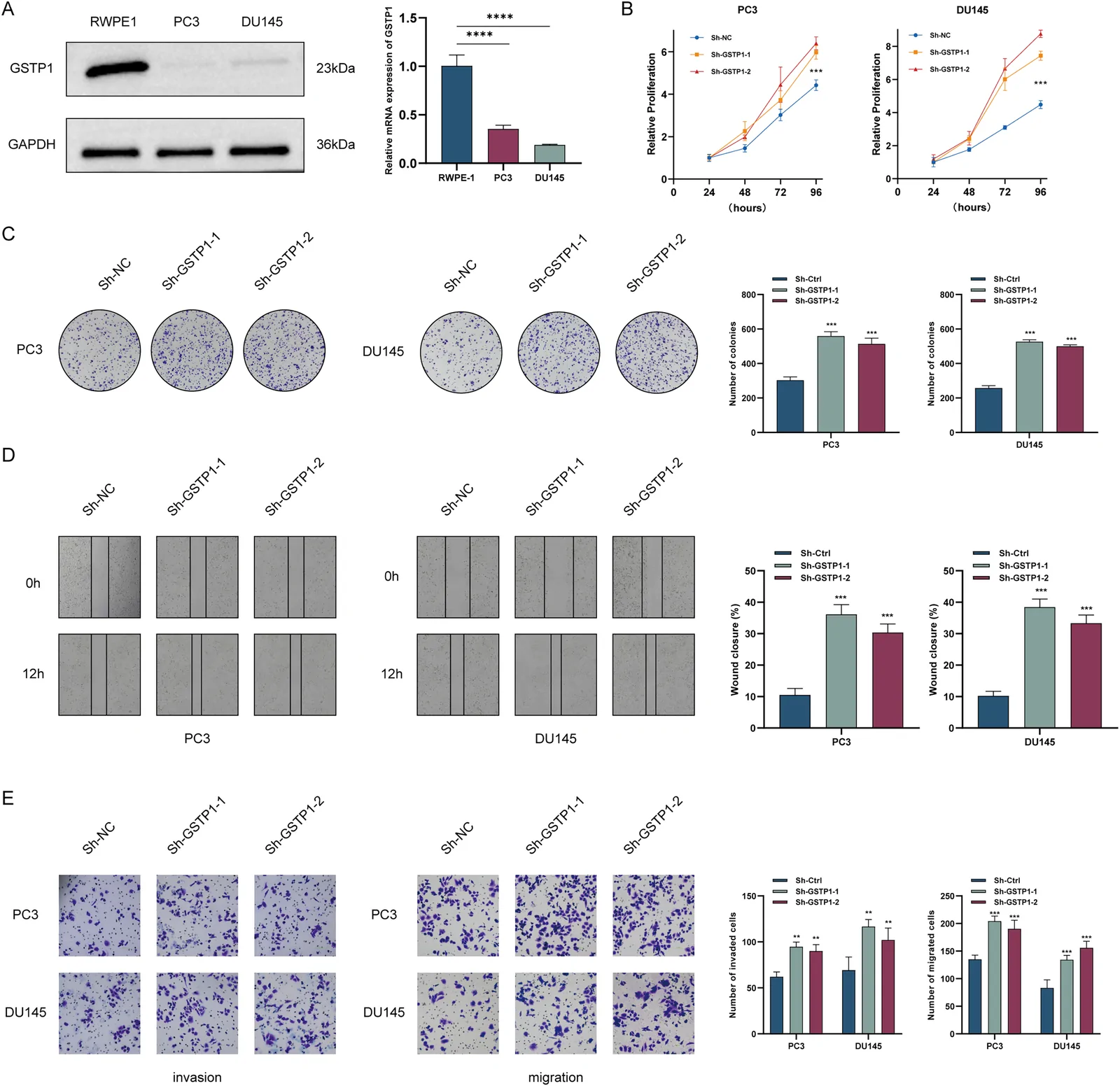
GSTP1 Suppresses PCa Cell line Proliferation and Metastasis. **(A)** Western blotting and qRT-PCR were used to validate GSTP1 expression in RWPE1, PC3, and DU145 cell lines, with GAPDH as the internal control (p < 0.05). **(B)** Proliferation assays (CCK-8) showed significantly elevated PCa cell proliferation after GSTP1 down-regulation compared to control groups (p < 0.05). **(C)** Colony formation assays revealed increased colony numbers and sizes in GSTP1-knockdown PCa cells (p < 0.05). **(D)** Wound healing assays demonstrated enhanced migratory capacity of PCa cells following GSTP1 suppression (p < 0.05). **(E)** Transwell assays visualized boosted invasion and migration cell counts in GSTP1-depleted groups (p < 0.05).

### 3.8 GSTP1 promotes PCa cell line aging progression

To investigate the role of GSTP1 in cellular aging, we evaluated SIPS through the measurement of SA-β-gal activity, and simultaneously conducted an analysis on the expression levels of pivotal senescence-associated proteins, such as p16 and p21, as well as DNA damage. Compared to the normal control group, the β-galactosidase-positive was significantly lower in the GSTP1 knockdown (**Figure 9A**). WB analysis showed reduced expression of cell cycle inhibitors (p16, p21) in shGSTP1 cells compared to sh-NC. The downregulation of p16 and p21, key mediators of cell cycle arrest, indicates that GSTP1 knockdown alleviates cell cycle blockade (**Figure 9B**). Next, we performed immunofluorescence (IF) staining for phosphorylated H2AX (γH2AX), a canonical marker for DNA double-strand breaks, in PC3 and DU145 transfected with either shNC, sh-GSTP1–1 and sh-GSTP1-2. In shNC-transfected cells, γH2AX exhibited characteristic nuclear foci with robust fluorescence intensity (**Figure 9C**). In contrast, sh-GSTP1–1 and sh-GSTP1–2 transfected cells displayed a marked reduction in both the number and intensity of γH2AX foci. These findings suggest that regulation of GSTP1 expression in the context of doxorubicin treatment can regulate the senescence phenotype in prostate cancer cells, thereby affecting their cell cycle and proliferation capacity.

**Figure 9:**
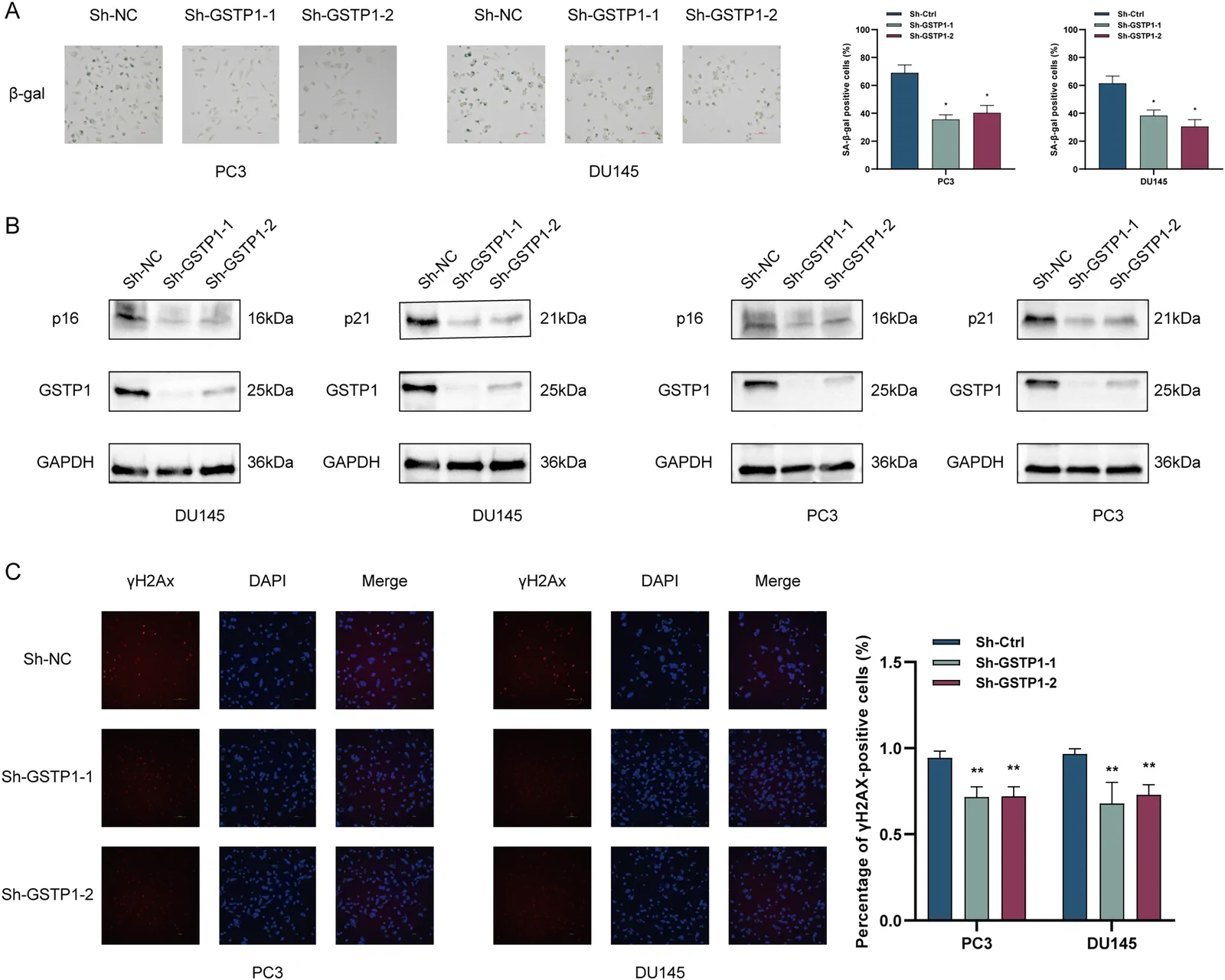
GSTP1 promotes PCa cell line aging progression **(A)** β-Galactosidase staining showing decreased senescence-associated β-galactosidase-positive cells in GSTP1-knockdown PCa cells.. **(B)** WB analysis revealing down-regulated expression of senescence-related markers p16 and p21 in GSTP1-depleted cells. **(C)** IF exhibited characteristic nuclear foci with fluorescence intensity of γH2AX.

## 4 Discussion

PCa continues to impose a significant global mortality burden on men, despite therapeutic advances (24). While insights into PCa genomics have catalyzed the development of molecularly stratified treatments (25), and numerous prognostic signatures exist (26, 27), the specific prognostic value of anti-androgen resistance-related gene sets remains underexplored. Furthermore, the functional impact of anti-aging mechanisms within the PCa context is poorly characterized. To bridge this gap, we aimed to construct a gene signature capable of facilitating diagnostic risk stratification and identifying patients for alternative therapies. This effort is grounded in the biology of cellular senescence—a fundamental tumor-suppressive program that arrests damaged cells (28). Senescent cells are identified by hallmark features such as morphological alteration, Lamin B1 loss, elevated SA-β-gal activity, chromatin remodeling, metabolic shifts, and SASP activation (29–31). Through transcriptomic analysis of 4 GEO databases, we identified that these aging-related genes were highly enriched in pathways related to PCa, identifying GSTP1 as a aging-related gene demonstrating exceptional diagnostic performance with a high AUC value. Multiple studies (32, 33) have shown that GSTP1 does not directly regulate the cellular aging phenotype in diseases. Instead, it acts as a key regulatory molecule in the oxidative stress process to mediate aging, thereby influencing the progression of the disease. However, it is not yet clear whether it can also mediate aging in prostate cancer. Subsequent analysis of gene expression patterns revealed that GSTP1 displayed pronounced differential expression patterns between PCa patients and healthy controls, suggesting its critical involvement in PCa pathogenesis and disease evolution. Furthermore, comparative immune profiling uncovered significant disparities in immune cell infiltration landscapes between PCa and healthy controls. The tumor immune microenvironment (TIME) is a critical determinant of cancer progression, where dynamic interactions between tumor and immune cells can facilitate immune evasion. Notably, tumor cells often hijack immune checkpoint pathways to suppress cytotoxic responses and enable uncontrolled growth (34). In response, immune checkpoint inhibitors (e.g., anti-CTLA-4, anti-PD-1/PD-L1) have been developed to reinvigorate antitumor immunity (35, 36). Mounting evidence further links the TIME composition to the development of therapy resistance, including resistance to anti-androgen therapies in PCa (37, 38). While classified as an immunologically “cold” cancer characterized by low tumor mutational burden, complex tumor microenvironment architecture, and limited antigenicity, the identification of immunotherapy-responsive PCa subpopulations remains a critical clinical priority (39). Thus, our findings reveal an association between GSTP1 expression and the immune cell composition of the PCa microenvironment, suggesting a potential link between GSTP1 and tumor–immune interactions. These observations provide a basis for further investigation of the role of GSTP1 in the immune microenvironment of PCa and its potential therapeutic relevance. Current evidence confirms the critical involvement of immune cell subpopulations in PCa pathogenesis (40, 41). The dual role of immune responses encompasses both protective effects and potential tissue-damaging consequences (42). Therefore, the immune-correlation analysis revealed GSTP1’s significant coordination with specific immune effector populations, particularly naïve B lymphocyte clusters and activated dendritic cell networks.

Guided by our bioinformatics analysis, we next sought to experimentally validate the pathogenic role of GSTP1 in PCa. For this purpose, the stable GSTP1-knockdown PC3 and DU145 cell line models were generated, serving as a cornerstone for dissecting GSTP1-depletion’s tumor-promoting mechanisms. Additionally, we also established a GSTP1-overexpression vector, but it did not attenuate of cellular proliferation (data not shown). Crucially, experimental re-expression of GSTP1 in PCa cell lines does not result in suppressed cell growth, indicating it does not function as a classical tumor suppressor gene in this context (43), consistent with our research findings. This observation has led to the hypothesis that GSTP1 may instead act as a “caretaker” gene within the prostate (44). Experimental validation revealed a significant reduction in GSTP1 expression following shRNA intervention. Functional validation confirmed that GSTP1 depletion unleashes a pro-tumorigenic phenotype. This included significantly elevated proliferative and clonogenic capacity, enhanced migratory and invasive potential, and the restoration of proliferative signaling—collectively suggesting its role in maintaining genomic integrity and restraining growth. However, GSTP1 depletion correlates with fewer γH2AX foci after doxorubicin treatment, which may reflect altered damage induction, repair or sensing (45–47). Relevant studies have shown that this phenomenon may be related to the activation of DNA damage and repair pathways and changes in the cell cycle. Notably, the pervasive epigenetic inactivation of GSTP1 in prostate carcinogenesis promotes cellular survival advantages and accrual of pro-carcinogenic DNA adducts following chronic oxidative stress exposure (48). Concomitant with these phenotypic shifts, we observed a marked attenuation of the senescence phenotype and diminished DNA damage response, pointing to its role in modulating the genotoxic stress threshold. This dual capacity to coordinate senescence and mitigate DNA damage response establishes GSTP1 as a potential mediator at the interface of aging and malignancy in PCa. These findings confirm GSTP1’s role in sustaining anti-aging, thereby facilitating PCa cell survival and invasiveness. By bridging computational predictions with experimental validation, our study highlights GSTP1 as both a differential expression marker and a candidate therapeutic target related to function in PCa.

While our study provides novel insights into the potential regulatory role of GSTP1 in PCa progression through integrated bioinformatics analysis and functional validation, several aspects warrant further investigation: First, while our analysis primarily leveraged publicly available transcriptomic datasets to identify GSTP1-related patterns, the relatively limited sample size in these cohorts necessitates cautious interpretation of the observed associations. Future studies should incorporate prospectively collected multi-institutional cohorts with standardized processing protocols to validate the observed GSTP1 expression patterns and their clinical correlations. Second, the mechanistic insights presented here are currently derived from cell culture models with restricted biological replicates and clinical tissue microarray analyses with expanded sample sizes are essential to confirm the translational relevance of our cellular findings. Meanwhile, the exclusive use of androgen-independent metastatic prostate cancer cell lines PC3 and DU145 in our study represents a limitation. Future investigations on GSTP1 should endeavour to include androgen-dependent cell lines such as LNCaP, so as to provide a more comprehensive elucidation of the role of this gene in the progression of prostate cancer. In the validation of aging-related phenotypes, we only examined P21, P16, β-galactosidase, and γ-H2AX, without assessing Lamin B1 or SASP factors, which should be supplemented in future studies. DNA damage analysis was limited to single-time-point γH2AX staining without comet assay or 8-OHdG detection, limiting mechanistic resolution. Finally, this study only revealed the correlation and functional phenotype of GSTP1 with the cellular aging pathway in the field of prostate cancer. The underlying mechanisms of this relationship remain to be explored. Collectively, these limitations emphasize that our current work represents a foundational step rather than a conclusive statement. Addressing these gaps through integrated multi-approaches, standardized preclinical modelling, and rigorously designed clinical studies will be essential to fully elucidate GSTP1’s role in PCa biology and its therapeutic potential.

## 5 Conclusions

In summary, our integrative analysis identified GSTP1 as an aging-associated gene. *In vitro* experiments showed that GSTP1 knockdown promotes proliferation, migration, invasion, and attenuates senescence-associated markers. Immune deconvolution revealed correlations between GSTP1 expression and several immune cell fractions, suggesting a possible link to the TME. These findings nominate GSTP1 as a promising biomarker and a candidate for functional studies, but further mechanistic and therapeutic investigations are warranted.

## Data Availability

The original contributions presented in the study are included in the article/**Supplementary Material**. Further inquiries can be directed to the corresponding authors.

## Supplementary material

Supplementary Figure 1 Standardized preprocessing procedures of raw data. **(A)** Box diagrams of GSE46602, GSE60329, GSE71781 and GSE79021 before calibration. **(B)** Box diagrams of GSE46602, GSE60329, GSE71781 and GSE79021 after calibration. **(C)** Principal component analysis of GSE46602, GSE60329, GSE71781 and GSE79021 before calibration. **(D)** Principal component analysis of GSE46602, GSE60329, GSE71781 and GSE79021 after calibration.

Supplementary Figure 2 Correlation plot visualizing correlations between GSTP1 and immune cells.

Supplementary Figure 3 Validation of GSTP1 knockdown efficiency.

Supplementary Table 1 Full list of aging-related genes obtained from the Aging Atlas database.

Supplementary Table 2 Complete Mendelian randomization results, including individual SNP estimates, heterogeneity statistics, and pleiotropy tests.

Supplementary Table 3 Complete gene lists identified by each individual method in the multi-tiered screening pipeline.
